# Methods used for indirect comparisons of systemic treatments for psoriasis. A systematic review

**DOI:** 10.1002/ski2.112

**Published:** 2022-04-23

**Authors:** Alexander Nast, Corinna Dressler, Christopher Schuster, Daniel Saure, Matthias Augustin, Kristian Reich

**Affiliations:** ^1^ Division of Evidence‐Based Medicine Department of Dermatology Venereology and Allergy Charité—Universitätsmedizin Berlin Berlin Germany; ^2^ Department of Dermatology Medical University of Vienna Vienna Austria; ^3^ Eli Lilly and Company Indianapolis Indiana USA; ^4^ Institute for Health Services Research in Dermatology and Nursing (IVDP) University Medical Center Hamburg‐Eppendorf (UKE) Hamburg Germany

## Abstract

**Background:**

Indirect comparisons (including network meta‐analyses [NMAs]) allow us to compare benefits and risks of multiple interventions for the same clinical condition when head‐to‐head comparisons are not feasible.

**Objective:**

To provide guidance to the clinical community on better understanding indirect comparison methods to help them to interpret their results by applying two quality standards to published indirect comparisons of systemic biologics for moderate to severe psoriasis.

**Methods:**

A systematic literature review (SLR) of published indirect comparisons of biologics for the treatment of moderate to severe psoriasis in adults was conducted. Data extraction was performed using a predefined subset of NICE TSD7 (National Institute for Health and Care Excellence Technical Support Document 7) checklist questions and methods used to perform each analysis were descriptively compared. Methodological quality of the SLR underlying each indirect comparison was assessed using AMSTAR 2 (A MeaSurement Tool to Assess systematic Reviews version 2).

**Results:**

Twenty‐two NMAs and four adjusted indirect comparisons (AICs) were identified. Although there were some similarities, for example, application of Bayesian random‐effects models, several important methodological aspects varied considerably across NMAs identified, for example, classes of drugs, number of treatments and studies included, reporting and handling of different doses, and reporting of both checks for and investigations of inconsistency. Methodological comparisons across AICs were limited by the small number. The quality of most underlying SLRs described, assessed as overall level of confidence in the results, was ‘critically low’.

**Conclusions:**

Understanding that there are different methodologies employed to answer differing research questions is key to helping clinicians to interpret the indirect evidence currently available in psoriasis.

## Graphical abstract

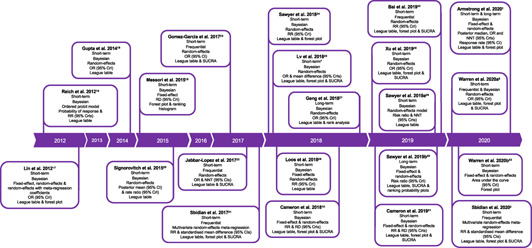

1

**What is already known about this topic?**

- Indirect comparison methods (including network meta‐analysis [NMAs]) can be applied to compare the benefits and risks of multiple interventions for the same clinical condition when head‐to‐head comparisons of all available treatments are not feasible. However, methods used to perform indirect comparisons to evaluate systemic therapies for psoriasis have become diversified over time, resulting in variability between the results of published analyses.

**What does this study add?**

- This review identified some similarities and several important methodological aspects that varied across NMAs in psoriasis, for example, classes of drugs, number of treatments and studies included, reporting and handling of different doses, and reporting of checks for and investigations of inconsistency. The quality, assessed as the overall level of confidence in the results, was ‘critically low’ for most indirect comparisons described.

**What are the clinical implications of this work?**

- For clinicians, understanding that there are different methodologies employed to answer differing research questions is key to helping them to interpret the evidence coming from indirect comparisons currently available in psoriasis.

## 2 INTRODUCTION

During the past decade, significant advances in the treatment of moderate to severe plaque psoriasis have resulted in a greater number of licenced systemic therapies, driving the need for relative efficacy and safety data to inform treatment decisions.^1^, ^2^ As a result, comparative efficacy analyses are increasingly of interest to clinicians and other healthcare professionals,^3^, ^4^ as well as payers and health technology assessment (HTA) decision‐makers. However, head‐to‐head studies of all licenced treatments are not feasible^3^, ^5^; therefore, methods used to perform indirect comparisons, including network meta‐analyses (NMAs), have evolved over time to meet this need.^6^

**FIGURE 1:**
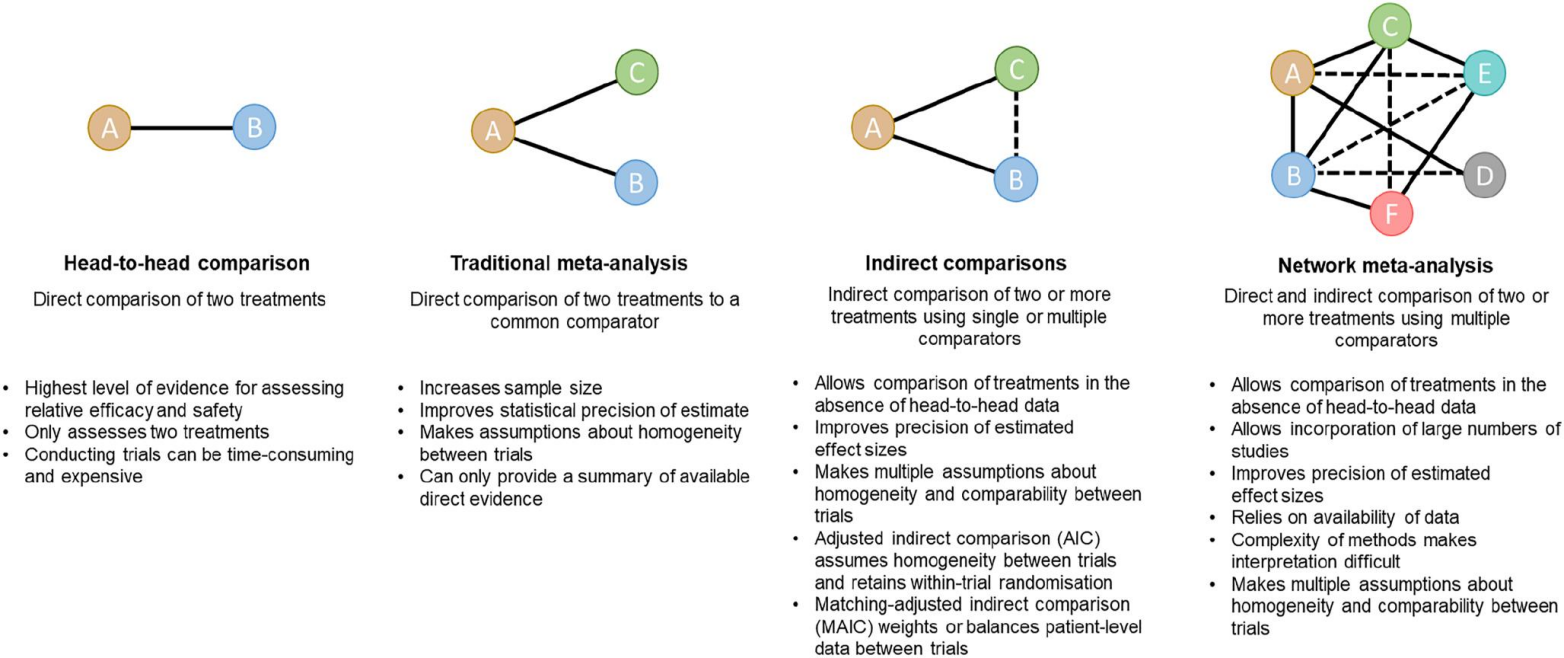
Methods for comparing relative efficacy. Solid lines represent evidence and dotted lines represent indirect evidence.

Adjusted indirect comparison (AIC) between two treatments is made through a common comparator,^7^ while NMAs use a combination of indirect and direct comparisons, usually derived from published randomised clinical trial (RCT) data, to compare more than two treatments in a single analysis (Figure 1).^3^ NMAs are increasingly accepted by national HTA bodies, as they can be useful for visualising large volumes of evidence, allow the relative effects of multiple interventions to be compared,^6^ and may provide findings key to the development of reimbursement recommendations.^8^, ^9^

While there is good consistency amongst clinical studies of systemic therapies for psoriasis, methods used to perform indirect comparisons have become diversified over time, resulting in variability between the results of published analyses.^10^, ^11^ NMA findings are directly linked to the methods used; therefore, audiences require an understanding of these methods to interpret the results.^6^ For those not trained in the complexities of the methodology, NMAs might be difficult to understand; there is a perception among some clinicians of ‘statistical trickery’ being used to achieve a particular conclusion.^3^, ^11^ Furthermore, while many clinical studies have some sort of acknowledged bias, scepticism remains towards NMAs due to the potential for the introduction of further bias.^2^ To ensure an NMA is easily accessible and understandable to the reader, a good publication must clearly present the methods employed, providing a statement of the decision problem, describing the scope of the research (including components of PICO [Population, Intervention, Comparison, and Outcome]), transparently and comprehensively reporting both findings (including network graphs) and the handling of heterogeneity and inconsistency, which occurs when the direct and indirect evidence conflict.

Quality standards, such as PRISMA‐NMA (Preferred Reporting Items for Systematic Reviews and Meta‐Analyses–Network Meta‐Analysis),^11^ NICE TSD7 (National Institute for Health and Care Excellence Technical Support Document 7) ‘Evidence synthesis of treatment efficacy in decision making: a reviewer's checklist’,^12^ AMSTAR 2 (A MeaSurement Tool to Assess systematic Reviews version 2)^13^ and PROSPERO (International Prospective Register of Systematic Reviews),^14^ have been developed to improve the transparency and accessibility of NMAs, and the systematic literature reviews (SLRs) undertaken to inform them.^15^

We undertook this study to determine whether we could develop guidance to help the clinical community better understand indirect comparison and NMA methodologies in order to assist them with interpreting the results of these types of analyses. We aimed to achieve this by systematically applying NICE TSD7 and AMSTAR 2 to published analyses of systemic biologics for moderate to severe plaque psoriasis and exploring their similarities and differences to identify key methodological aspects to highlight.

## 3 METHODS

### 3.1 Search strategy

A systematic literature review was conducted to identify published indirect comparisons, including NMAs, of biologics for the treatment of moderate to severe plaque psoriasis in adults (≥18 years), with the aim of summarising the methods used to undertake the analyses. The study protocol is registered at PROSPERO (CRD42020163081; available at: https://www.crd.york.ac.uk/prospero/display_record.php?ID=CRD42020163081). Amendments to the protocol are described in the supplementary material. Eleven electronic databases (accessed through Ovid) were searched until 26 March 2020 (for details see supplementary material).

### 3.2 Study selection

**FIGURE 2:**
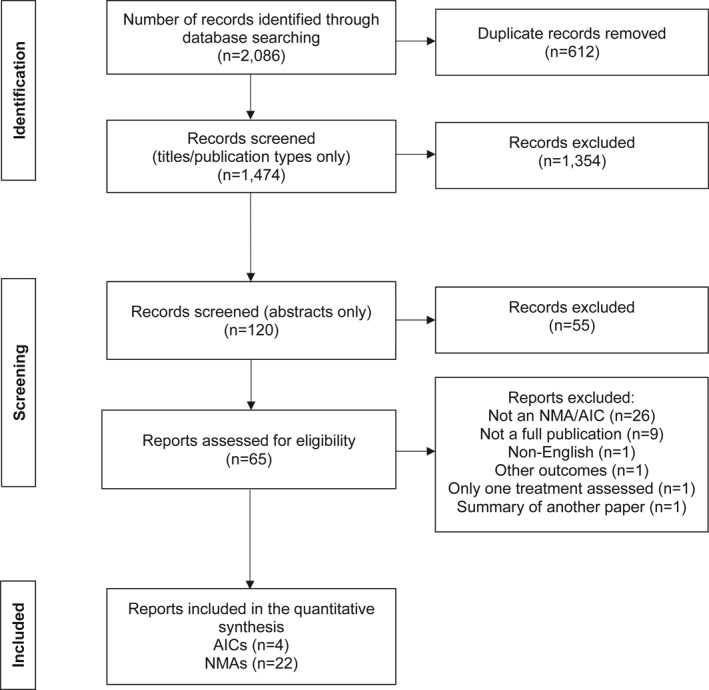
Literature search flow diagram. AIC, adjusted indirect comparison; NMA, network meta‐analysis

Study inclusion criteria are outlined in Table 1. The screening process was separated into three stages (Figure 2). At each stage, publications identified in the literature search were screened against the eligibility criteria. Screening was carried out by one independent reviewer and checked by selected authors (C.S. and D.S.).

**TABLE 1:** Eligibility criteria for indirect comparisons to be included in systematic review

Study characteristic	Inclusion criteria
Study design	Adjusted indirect comparison, matching‐adjusted indirect comparison, network meta‐analysis
Patient population	Adult patients with moderate to severe psoriasis
Intervention	Any biologic treatment for psoriasis
Comparator	Placebo or any systemic treatment for psoriasis
Outcomes	Efficacy, safety
Publication type	Journal article
Language	English

### 3.3 Data extraction and synthesis

Data extraction was performed using an Excel template developed based on a predefined subset of the NICE TSD7 checklist^12^ questions applicable to indirect comparisons in psoriasis; handling/pooling of different drug doses was added as an additional item (for details see Table S1). Data extraction was carried out by two reviewers and checked for inconsistencies by a third reviewer; inconsistencies were resolved through discussion.

Methods used to perform each identified indirect comparison were descriptively compared within the following three categories: (1) methods of analysis and presentation, (2) definition of decision problem (i.e., a description of the researcher's approach to the research question, including a description of the population, intervention, comparator(s), outcomes and any special considerations) and (3) issues specific to network synthesis.

### 3.4 Quality and risk of bias assessment

The methodological quality of the SLR underlying each identified indirect comparison was assessed using the 2017 AMSTAR 2 critical appraisal tool.^13^ AMSTAR 2, a 16‐item instrument that classifies the level of confidence in the results of an SLR of healthcare interventions as ‘high’, ‘moderately low’, ‘low’, or ‘critically low’, based on seven critical and nine non‐critical domains, was selected to assess the quality of the indirect comparisons identified in this study. Each publication was independently assessed by two reviewers and a consensus decision for each item and the overall methodological quality of each publication was mediated by a third reviewer.

## 4 RESULTS

Overall, the literature review identified 1,474 potentially relevant analyses following deduplication (Figure 2). A total of 26 analyses in moderate to severe plaque psoriasis met the inclusion criteria.

### 4.1 Data synthesis

#### 4.1.1 Methods of analysis and presentation

**FIGURE 3:**
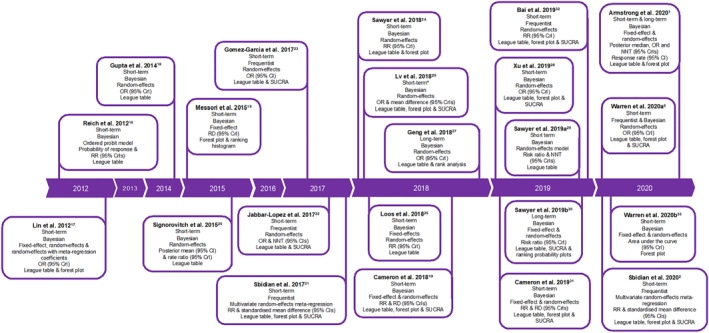
Meta‐analytic methods used to undertake network meta‐analyses in chronological order by date of publication (*N* = 22). *NMA also included two long‐term (>40‐week studies). CI, confidence interval; CrI, credibility interval; NNT, number needed to treat; OR, odds ratio; RD, risk difference; RR, relative risk; SUCRA, surface under the cumulative ranking curve

Analytical methods used to undertake 22 identified NMAs are described in chronological order by date of publication in Figure 3.^1^, ^2^, ^5^, ^10^, ^16^, ^17^, ^18^, ^19^, ^20^, ^21^, ^22^, ^23^, ^24^, ^25^, ^26^, ^27^, ^28^, ^29^, ^30^, ^31^, ^32^, ^33^ Most NMAs evaluated only short‐term outcomes (19/22%; 86%): two analyses assessed long‐term outcomes and one assessed both short‐ and long‐term outcomes. Bayesian statistical analysis was most commonly used (17/22 NMAs; 77%), and random‐effects modelling was most often presented (20/22 NMAs; 91%), with a trend towards both random‐effects and fixed‐effect model results presentations in recent years. Adjustment for baseline risk (i.e., placebo response) was considered in several analyses. Reported effect measures (e.g., risk ratio, odds ratio, risk difference, mean difference, etc.) varied considerably. However, most NMAs (21/22; 95%) provided treatment rankings to be interpreted alongside actual numerical differences between therapies to allow conclusions on the clinical relevance of reported outcomes. The league table was the most common method of presenting these treatment rankings (19/21; 90%), although approximately half of the NMAs that provided treatment rankings presented surface under the cumulative ranking curve (SUCRA) values (10/21; 48%), with a trend towards increased use of SUCRA in more recent years.

Four AICs were also identified. Methods of analysis and presentation (Table S2), as well as decision problem definitions (Table S4) are described in supplementary materials.

#### 4.1.2 Definition of decision problem

A comparison of methods with respect to definition of the decision problem for the 22 NMAs identified is presented in Table S3. The target population was clearly defined as adult patients with moderate to severe psoriasis for all NMAs, with similar inclusion criteria across the analyses; 59% (13/22) of NMAs included patient populations with concomitant psoriatic arthritis, while one analysis set concomitant psoriatic arthritis as an exclusion criterion. All NMAs were based on data from RCTs of systemic therapies; however, treatments included in each analysis varied greatly. The number of treatments considered ranged from 3 to 19. Nine analyses focussed entirely on biologics, five included biologics (one of which included biosimilars) and small molecules, four analyses included biologics and conventional systemics and four included all three treatment classes. Some variation was also identified in the reporting and handling of treatment doses. The majority of NMAs included European Medicines Agency (EMA)‐ and/or Food and Drug Administration (FDA)‐approved doses (19/22); however, one analysis specified ‘approved doses’ only, one combined treatment results across all doses but included a subgroup analysis of FDA‐approved doses only and one provided no dose information but pooled treatments by class.

All NMAs reported efficacy findings with one exception; Messori et al. 2015^19^ described safety findings only. The majority of NMAs reported patients achieving 75% and 90% improvement in Psoriasis Area Severity Index (PASI75 and PASI90) efficacy outcomes (19/22 each; 86%); patients achieving 50% and 100% improvement in PASI (PASI50 and PASI100) were each included in ten and nine analyses, respectively. Limited quality of life data were reported: only eight NMAs described Dermatology Life Quality Index (DLQI) outcomes. Safety data were included in only ten NMAs: relatively detailed safety data were reported by Lv et al. 2018^25^ and Loos et al. 2018,^26^ whereas limited safety data (e.g., adverse events [AEs], infectious AEs, serious adverse events [SAEs], and/or study discontinuation or withdrawal due to AEs) were documented in the other eight analyses.

Most NMAs did not include a discussion on treatment effect modifiers (16/22; 73%). Risk of bias was usually discussed (15/22; 68%) and considered to be low in most studies. Potential risks considered were either due to imbalances in patient and outcome characteristics, or small study effects (i.e., publication bias).

#### 4.1.3 Issues specific to network synthesis

A comparison of methods specific to network synthesis for the 22 NMAs identified is presented in Table S5. Graphical representations of evidence networks, which are crucial to understanding the analyses, were displayed for 20/22 NMAs (91%). The networks of evidence were connected in all 20 of these analyses (100%).

Adequate checks for inconsistency were reported in 12/22 NMAs (55%) using various methods, including node‐splitting analyses, loop‐specific and side‐splitting approaches, net heat plots, a random‐effects unrelated mean effects model and the two‐stage Bucher method. Checks for inconsistency were not described in 9/22 NMAs (41%) and only descriptively outlined in one NMA. Overall, inconsistency was not reported to be an issue. Heterogeneity was detected mainly in disease severity across RCT populations and investigated using standard subgroup analysis approaches.

### 4.2 Quality and risk of bias assessment

Of the 26 analyses identified, based on the number of critical and non‐critical items in each analysis assessed using AMSTAR 2,^13^ the overall level of confidence in the results was ‘high’ for two NMAs, ‘low’ for one NMA and ‘critically low’ for the remaining 23 analyses (Table 2 and Figure S1). Proportions of analyses assessed as having each critical and non‐critical flaw are presented in Table 2 (and Figures S2a and S2b, respectively). The only critical criterion met by more than 50% of the analyses was ‘use of appropriate methods for statistical combination of results of meta‐analysis’. The three critical criteria related to the assessment of risk of bias in individual studies in the review, accounting for risk of bias in primary studies when discussing results, and investigation of small study bias and its likely impact on results, were met by only 12, 5 and 6 of the 22 identified analyses (46%, 19% and 23%), respectively. The only non‐critical criteria met by more than 50% of the analyses were: ‘research questions and inclusion criteria for review included components of PICO’; ‘satisfactory explanation for, and discussion of, any heterogeneity observed in results of review provided’; and ‘potential sources of conflict of interest, including any funding received for conducting review, reported’.

**TABLE 2:** AMSTAR 2 critical and non‐critical domain assessment results and overall confidence in results of adjusted indirect comparisons and network meta‐analyses based on AMSTAR 2 assessment of underlying systematic literature reviews (*N* = 26)

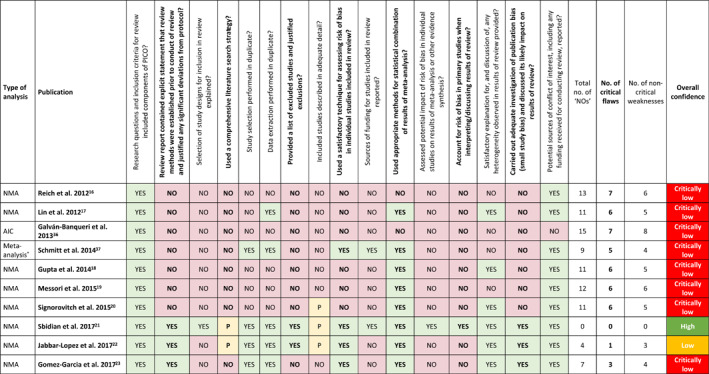
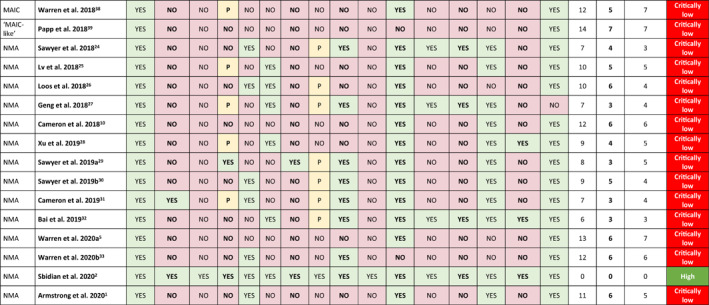

*Note*: Critical flaws are in bold font; non‐critical flaws are in regular font.

Abbreviations: AIC, adjusted indirect comparison; MAIC, matching‐adjusted indirect comparison; NMA, network meta‐analysis; PICO, Population, Intervention, Comparison, and Outcome.

^a^Schmitt et al. 2014^37^—direct and indirect comparative efficacy was assessed by random effects meta‐analysis of risk differences.

## 5 DISCUSSION

This study identified 26 indirect comparisons of systemic treatments for moderate to severe plaque psoriasis and compared the methods and quality of these analyses using NICE TSD7 and AMSTAR 2 assessment tools, respectively. Methods applied across all indirect comparisons varied; although Bayesian random‐effects models were applied in most NMAs, there was considerable variation in other methodological aspects, such as classes of drugs, number of treatments and studies included in each analysis (with increases year on year in line with licenced availability), reporting and handling of different doses, as well as both checks for and investigations of inconsistency. The quality of the SLRs underlying the indirect comparisons, assessed as the overall level of confidence in the results, was ‘critically low’ for 23 of the 26 indirect comparisons identified. Below we outline a set of key learnings and practical considerations to provide guidance to the clinical community on understanding these methods to facilitate interpretation of their results (Table 3). Interestingly, another recently published assessment of the quality and coherence of NMAs of biologics in plaque psoriasis was broadly aligned with our findings, recommending treatment decisions should be made using high quality, up‐to‐date NMAs with assumptions relevant to clinical practice.^40^

**TABLE 3:** Key learnings from this study and practical considerations for clinicians

No.	Key learning	Practical consideration
1.	Although they may appear to address similar research questions, all indirect comparisons differ in terms of specific characteristics (e.g., treatments and dose regimens included, outcomes reported, etc.).	Consider what you want to know (e.g., am I only interested in licenced doses or unlabelled dose efficacy data, a specific subset of biologics or a picture of the whole landscape, efficacy on specific endpoints?) and look for the NMA fulfilling all necessary quality standards that best answers your question
2.	Effect measures reported and the way in which results are presented (e.g., treatment rankings) vary across indirect comparisons and can be misleading.	Treatment rankings aim to provide a clear answer to the question ‘which treatment is performing best?’; however, it should be noted that treatment rankings are usually derived based on one endpoint only. Even more importantly, always check the magnitude of the effect difference across treatments to get a better understanding of the ranking (e.g., treatments 1 and 2 may be more similar in terms of efficacy than treatments 2 and 3)
3.	Indirect comparisons may exhibit methodological inadequacies, such as lack of graphical representations of evidence networks, absence of discussion of treatment effect modifiers, lack of assessment of or adjustment for risk of bias, or inadequate checks for consistency and/or adjustments made in response to identified inconsistencies	Inconsistency is one of the key issues with NMA. Cross‐check NMA findings with the results of head‐to‐head trials to get a better understanding of the findings and their plausibility if the NMA publication does not provide such a comparison. Always interpret NMA findings alongside available head‐to‐head trial data
4.	There is a need for improved quality across all indirect comparisons, including NMAs	There is a need to balance quality, transparency and scientific credibility of indirect comparisons with the practicalities of day‐to‐day clinical practice. Be aware of the strengths and limitations of indirect comparisons and give appropriate weight to their findings in the context of the bigger picture and all available information

Abbreviation: NMA, network meta‐analysis.

Key learning # 1 **Although they may appear to address similar research questions, all indirect comparisons differ in terms of specific characteristics (e.g., treatments and dose regimens included, outcomes reported, etc.).**

Some aspects of the decision problem definitions were very similar for most indirect comparisons, as highlighted via the NICE TSD7 analysis. Target patient populations were clearly described and based on similar disease definitions across the analyses. Efficacy outcomes were fairly consistent, as they are in clinical trials for psoriasis, with PASI75 and PASI90 outcomes reported for the majority of indirect comparisons.

Important differences identified between these indirect comparisons, highlighting areas of focus for harmonisation, included the selection of treatments or treatment classes. There was considerable variability between analyses with respect to inclusion of comparators, although networks became more comprehensive over time with more treatments becoming available, thereby increasing the complexity of the models required to run the analyses. There were also differences between analyses with respect to the reporting and handling of doses (e.g., multiple licensed doses per product were sometimes pooled, sometimes analysed separately).

Consideration of quality of life data was limited across all analyses, potentially highlighting a gap in the data currently available from RCTs in psoriasis, or simply a need to include all available data in indirect comparisons moving forward in order to gain a complete perspective on the true relative value of treatments for moderate to severe psoriasis.

Key learning # 2 **Effect measures reported and the way in which results are presented (e.g., treatment rankings) vary across indirect comparisons and can be misleading.**

There was limited consistency across the indirect comparisons with respect to the effect measure(s) reported, with risk ratios, odds ratios, risk differences, mean differences or posterior means. The selection of appropriate effect measures is determined by the type of outcomes reported (e.g., binary vs. continuous) and somewhat by the availability of sufficient data. Multiple effect measures were commonly reported per analysis.

Traditional league tables were most commonly used to present results and treatment rankings, often alongside forest plots; however, we observed an increased use of SUCRA to present treatment rankings in recent years. Overall, ranking approaches should be viewed with caution, as they are typically based on a single outcome measure, presented without accompanying measures of uncertainty^34^, ^35^ or differences in ranking may be based on modest numerical differences in clinical efficacy or safety.^6^

Key learning #3 **Indirect comparison publications may exhibit methodological or reporting inadequacies, such as lack of graphical representations of evidence networks, absence of discussion of treatment effect modifiers, lack of assessment of or adjustment for risk of bias or inadequate checks for consistency and/or adjustments made in response to identified inconsistencies.**

In this review, the networks of evidence were connected for all NMAs identified and graphical representations of evidence networks, crucial to understanding the analyses, were displayed for most NMAs. However, several consistent methodological inadequacies were identified; there was no discussion on treatment effect modifiers (i.e., patient characteristics that influence treatment outcomes) in most publications and, although risk of bias was discussed in 15 of 22 NMAs and generally considered low, adjustments and adequate justification for those adjustments were only made in six NMAs. We observed that adjustments for baseline risk (i.e., placebo response) were carried out more frequently over time, which is important as lack of adjustment for cross‐trial differences can lead to different clinical interpretations of findings. Any indirect comparison approach can be criticised; therefore, discussions on bias are necessary to ensure complete transparency to allow the reader to assess the risks and interpret the results with the appropriate caveats.

Furthermore, to move towards a methodological gold standard in psoriasis, significant improvements are required with respect to the reporting of checks for inconsistency, methods used to do so and adjustments made to address any inconsistencies identified. Inconsistencies were not reported to be explored in almost half of the NMAs identified in this review, and, if checked, methods were sometimes not described. Adjustments for identified inconsistencies were rarely mentioned.

Key learning #4 **There is a need for improved quality across all indirect comparisons, including NMAs.**

Overall, our analysis based on AMSTAR 2 highlighted the need for improved quality of methods and reporting across the SLRs carried out to inform indirect comparisons, including NMAs, in psoriasis. According to this tool, only 2 of the 26 indirect comparisons identified were of ‘high’ quality (the second being an update of the first NMA), meaning the reader could be confident in the results; all other indirect comparisons were of ‘low’ or ‘critically low’ quality. However, ‘high quality’ analyses do not always provide consistent answers. Application of checklists during the planning and conduct of an indirect comparison, such as AMSTAR 2 or NICE TSD7, is generally recommended to improve quality, and there are reporting guidelines (e.g., PRISMA NMA).^11^

### 5.1 Limitations

We investigated only a subset of NICE TSD7 checklist questions (i.e., those believed to be relevant to this analysis); however, the list of pre‐specified characteristics compared may be incomplete, with important characteristics missing. The results of our AMSTAR 2 appraisal may be influenced by the fact that some NMA publications may not include full details of the underlying SLR carried out to inform the analysis.

## 6 CONCLUSIONS

Indirect comparisons, including NMAs, can provide valuable indirect evidence of the short‐term efficacy of available treatment options for moderate to severe psoriasis using currently available data, especially the assessment of high levels of skin clearance (PASI75 and 90). However, current differences in indirect comparison methods identified in this study highlight the need for clinicians to have an understanding of these methods to facilitate interpretation of the results. Our main objective was to provide guidance for the clinical community to find their way around the plethora of published indirect evidence in psoriasis in order to help with their interpretation of the results and support evidence‐based clinical decision making in this chronic disease.

## CONFLICT OF INTEREST

AN and CD declare no conflicts of interest. CS is an employee of Eli Lilly and Company. DS was an employee and stockholder of Eli Lilly and Company at the time the work was conducted. MA has received research grants from AbbVie, Almirall, Beiersdorf, Eli Lilly, Galderma, Incyte, LEO, Menlo, MSD, Novartis, Pfizer, Regeneron, Sanofi‐Genzyme and Trevi; served as a consultant for AbbVie, Almirall, Beiersdorf, Eli Lilly, Galderma, Incyte, LEO, Menlo, MSD, Novartis, Pfizer, Regeneron, Sanofi‐Genzyme and Trevi; and as a lecturer for AbbVie, Almirall, Beiersdorf, Eli Lilly, Galderma, Incyte, LEO, Menlo, MSD, Novartis, Pfizer, Regeneron, Sanofi‐Genzyme and Trevi. KR has served as advisor and/or paid speaker for and/or participated in clinical trials sponsored by Abbvie, Almirall, Amgen, Boehringer Ingelheim, Bristol‐Myers Squibb, Celgene, Forward Pharma, Gilead, Galderma, Janssen‐Cilag, Kyowa Kirin, Leo, Eli Lilly and Company, Medac, Novartis, Ocean Pharma, Pfizer, Sanofi, UCB. KR is co‐founder of Moonlake Immunotherapeutics.

## AUTHOR CONTRIBUTIONS

Alexander Nast, Corinna Dressler, Christopher Schuster and Daniel Saure contributed to the study conception/design. Data acquisition and analysis were performed by Daniel Saure. All authors were involved in interpretation of data for the work, revised the manuscript critically for important intellectual content, and have read and approved the final version.

## Supporting information

Supplementary Material S1

## Data Availability

Data sharing is not applicable to this article as no new data were created or analysed in this study.
