# Supplementary material for: Methods used for indirect comparisons of systemic treatments for psoriasis. A systematic review
Source: Skin Health Dis. 2022 Apr 23;3(1):e112. doi: 10.1002/ski2.112 (PMC9892472; doi:10.1002/ski2.112)
Supplement: Supplementary file 1 — Supplementary Material S1 [file SKI2-3-e112-s001.pdf]

Supplementary material

Methods

Amendments to protocol

The following amendments were made to the protocol after initial registration: the title was changed from ‘umbrella’ literature review to ‘systematic’ literature review, as the focus of the project changed; the date of the updated search was added (26 March 2020); 'English' and 'Humans' were added as filters to the search string; mention of the NICE TDS7 checklist was added; further details were added to the data extraction, risk of bias assessment and data synthesis strategy sections; and the stages of the review (all started and completed) were updated.

Search strategy

The following electronic databases were accessed through Ovid: MEDLINE®, MEDLINE® In-process, E-pubs ahead of print, Embase, Cochrane Database of Systematic Reviews, American College of Physicians Journal Club, Database of Abstracts of Reviews of Effects, Cochrane Central Register of Controlled Trials, Cochrane Methodology Register, HTAs and National Health Service Economic Evaluation Database. Only English-language full-text articles were included.

The final search string was as follows: ‘(psoriasis) [Title/abstract] AND (meta-analysis OR meta analysis OR network meta-analysis OR network meta analysis OR indirect comparison*) [All fields]’

Study selection

At each stage publications identified in the literature search were screened against the eligibility criteria (Table 1). Abstracts of publications selected at the first stage were then screened for eligibility. Finally, full texts of publications identified at the second stage were checked for eligibility.

Data extraction and synthesis

Table S1. List of items extracted from publications identified in the systematic review

Extracted items based on predefined subset of questions from the NICE TSD7 checklist[1]
• Follow-up and treatment duration<br>• Statistical analysis (e.g., method, imputation of data, sensitivity analyses, risk of bias assessment)<br>• Effect measure(s)<br>• Presentation of results and/or treatment ranking<br>• Target population and population demographics<br>• Treatment dosing and handling of different dosages<br>• Modifiers of treatment effect (if available)<br>• Individual study inclusion/exclusion criteria and studies eligible for inclusion (e.g., Phase II/III/IV, placebo-controlled or head-to-head, treatments)
**Items based on efficacy versus placebo or active comparator**
• Patients achieving PASI75/90/100<br>• Physician's Global Assessment score<br>• Investigator's Global Assessment score<br>• Absolute PASI values ≤1, ≤2, ≤3, ≤5<br>• Mean change in PASI<br>• Patient reported-outcomes relating to quality of life (DLQI)
**Items based on safety versus placebo or active comparator**
• Serious adverse events<br>• Treatment adverse events<br>• Adverse events of special interest<br>• Discontinuation due to adverse events

DLQI, Dermatology Life Quality Index; NICE TSD7, National Health and Care Excellence Decision Support Unit Technical Support Document 7; PASI, Psoriasis Area and Severity Index; PASI50, 75, 90 & 100; Psoriasis Area and Severity Index 50%, 75%, 90% & 100% reduction from baseline.

The preferred statistical representation of data for both efficacy and safety outcomes are odds ratios or risk ratios, with 95% credible or confidence intervals, and surface under cumulative ranking (SUCRA) estimates or p-values.

Quality and risk of bias assessment

Consensus assessments for all 16 items for all identified publications were pooled and inter-rater reliability (IRR) was calculated (according to Ranganathan et al. 2017)[2] for all items, critical items and non-critical items. The IRR, i.e., Cohen's kappa, for each assessment was calculated using Microsoft Excel 2016 and confirmed using SPSS version 20, with a p value of ≤0.05 considered statistically significant. Kappa statistic cut-offs were interpreted as per the literature: ≤0.20 = slight agreement; 0.21–0.40 = fair agreement; 0.41–0.60 = moderate

agreement; 0.61–0.80 = substantial agreement; 0.81–0.99 = near-perfect agreement; and 1.00 = perfect agreement.[2]

Results

Data synthesis

1. Methods of analysis and presentation

Four adjusted indirect comparisons (AICs) were identified, each evaluating short-term outcomes (Table S2).[3–6] One analysis used the Bucher method[7] to undertake an AIC, whereas another employed the same Bucher method, along with a modified version of the Signorovitch method (balanced or weighted per treatment arm separately vs. overall as with the original method),[8] to perform a matching-adjusted indirect comparison (MAIC). A third analysis balanced baseline characteristics based on propensity score matching and model fit in an “MAIC-like" approach, and the fourth assessed direct and indirect comparative efficacy by random-effects meta-analysis of risk differences. Each analysis reported different single or multiple effect measures, including risk difference, absolute risk reduction, odds ratio (versus placebo or common active comparator) and propensity score-weighted proportions of patients achieving outcomes.

Table S2. Comparison of adjusted indirect comparisons with respect to methods of analysis based on selected National Institute for Health and Care Excellence Technical Support Document 7 checklist questions (N=4)

NICE TSD7 question	Galván-Banqueri et al. 2013[3]	Schmitt et al. 2014[4]	Warren et al. 2018[5]	Papp et al. 2018[6]
**Short- vs. long-term**	Short-term (12–24 weeks)	Short-term (8–16 weeks)	Short-term (12 weeks)	Short-term (12 weeks)
**Statistical method**	AIC (Bucher method)	Random-effects meta-analysis	MAIC (Bucher method + modified version of Signorovitch method)	“MAIC-like” approach (baseline characteristics balanced based on propensity score matching and model fit)
**Effect measure(s)**	Absolute risk reduction (95% CI)	Risk difference (95% CI)	Risk difference & odds ratio (vs. placebo or common active comparator) (95% CIs)	Propensity score-weighted proportions of patients achieving outcomes
**Method of presentation of results**	Descriptive table	League table & forest plot	Forest plot	Histogram & descriptive table

AIC, adjusted indirect comparison; CI, confidence interval; MAIC, matching-adjusted indirect comparison; N/A, not applicable; NICE TSD7, National Institute for Health and Care Excellence Technical Support Document 7.

2. Definition of decision problem

Table S3. Comparison of network meta-analysis methods with respect to definition of the decision problem based on selected National Institute for Health and Care Excellence Technical Support Document 7 checklist questions (N=22)

NICE TSD7 question	Reich et al. 2012[9]	Lin et al. 2012[10]	Gupta et al. 2014[11]	Messori et al. 2015[12]	Signorovitch et al. 2015[13]	Sbidian et al. 2017[14]	Jabbar-Lopez et al. 2017[15]	Gómez-Garcia et al. 2017[16]	Sawyer et al. 2018[17]	Lv et al. 2018[18]	Loos et al. 2018[19]
**Has the target population for the decision been clearly defined?**	Yes, adult patients with moderate-to-severe psoriasis	Yes, patients (>18 years old) with moderate-to-severe psoriasis who were candidates for phototherapy or systemic therapy	Yes, patients (>18 years old) with moderate-to-severe psoriasis, with ≥10% PASI at baseline, and not undergoing concomitant psoriasis therapy during the study period	Yes, patients with moderate-to-severe psoriasis	Yes, patients with moderate-to-severe psoriasis	Yes, patients (>18 years old) with moderate-to-severe psoriasis or psoriatic arthritis with concomitant moderate-to-severe psoriasis	Yes, patients (>18 years old) with moderate-to-severe psoriasis	Yes, patients (>18 years old) with moderate-to-severe psoriasis	Yes, patients with moderate-to-severe psoriasis (concomitant psoriatic arthritis were excluded)	Yes, patients with psoriasis	Yes, adult patients with moderate-to-severe psoriasis (including concomitant psoriasis types or psoriatic arthritis)
**Have all the appropriate treatments in the decision been identified?**	No<br>Adalimumab<br>Efalizumab*<br>Etanercept<br>Infliximab<br>Ustekinumab	No<br>Adalimumab<br>Alefacept*<br>Etanercept<br>Infliximab<br>Ustekinumab	No<br>Adalimumab<br>Alefacept*<br>Etanercept<br>Infliximab<br>Methotrexate<br>Ustekinumab	No<br>Adalimumab<br>Etanercept<br>Ustekinumab	No<br>Adalimumab<br>Efalizumab*<br>Etanercept<br>Infliximab<br>Ustekinumab	Yes<br>Acitretin<br>Adalimumab<br>Alefacept*<br>Apremilast<br>Brodalumab<br>Certolizumab<br>Ciclosporin<br>Etanercept<br>FAEs<br>Guselkumab<br>Infliximab<br>Itolizumab*<br>Ixekizumab<br>Methotrexate<br>Ponesimod*<br>Secukinumab<br>Tildrakizumab<br>Tofacitinib*<br>Ustekinumab	No<br>Adalimumab<br>Etanercept<br>Infliximab<br>Ixekizumab<br>Methotrexate<br>Secukinumab<br>Ustekinumab	No<br>Adalimumab<br>Etanercept<br>Infliximab<br>Secukinumab<br>Ustekinumab	No<br>Adalimumab<br>Apremilast<br>Brodalumab<br>Etanercept<br>Infliximab<br>Ixekizumab<br>Secukinumab<br>Ustekinumab	Yes<br>Adalimumab<br>Alefacept*<br>Briakinumab*<br>Brodalumab<br>Efalizumab*<br>Etanercept<br>Golimumab<br>Guselkumab<br>Infliximab<br>Itolizumab*<br>Ixekizumab<br>Methotrexate<br>Secukinumab<br>Tildrakizumab<br>Ustekinumab	No<br>Adalimumab<br>Apremilast<br>Brodalumab<br>Etanercept<br>Infliximab<br>Ixekizumab<br>Secukinumab<br>Ustekinumab

**Has information been provided, how different doses were integrated into the analysis?†**	Yes<br>EMA-approved doses only (per SmPC)<br>Analysed dosages separately	Yes<br>FDA-approved doses<br>Analysed dosages separately	Yes<br>FDA-approved doses<br>Multiple dosages were pooled	Yes<br>EMA-approved doses only (per SmPC)<br>Analysed dosages separately	Yes<br>EMA- and FDA-approved doses<br>Analysed dosages separately	Yes<br>FDA-approved doses<br>Multiple dosages were pooled	Yes<br>Combined across all treatment doses<br>(Subgroup analysis of FDA-approved doses only was performed)	Yes<br>EMA- and FDA-approved doses only<br>Analysed dosages separately	Yes<br>EMA- and FDA-approved doses only<br>Analysed dosages separately	No<br>No dosing information provided – treatments pooled by class	Yes<br>FDA-approved doses only<br>Multiple dosages were pooled
**Have all trials reporting relevant outcomes been included?**	No<br>**Efficacy:** PASI50, 75 & 90<br>**No QoL**<br>**No safety**	No<br>**Efficacy:** PASI50, 75 & 90<br>**No QoL**<br>**No safety**	No<br>**Efficacy:** PASI75<br>**No QoL**<br>**No safety**	No<br>**No efficacy**<br>**No QoL**<br>**Safety:** any infectious AE, SAE	No<br>**Efficacy:** PASI50, 75 & 90<br>**No QoL**<br>**No safety**	No<br>**Efficacy:** PASI75 & 90, PGA (0,1)<br>**QoL:** DLQI (0,1)<br>**Safety:** AE, SAE	No<br>**Efficacy:** minimal residual activity/PASI >90/PGA (0,1) & PASI75<br>**QoL:** mean change in DLQI<br>**Safety:** d/c due to AE	No<br>**Efficacy:** PASI75 & 90, IGA/PGA/ sPGA (0,1)<br>**QoL:** DLQI (0,1)<br>**Safety:** >1 AE, infectious AE, SAE, d/c due to AE	No<br>**Efficacy:** PASI50, 75, 90 & 100<br>**No QoL**<br>**No safety**	No<br>**Efficacy:** PASI50, 75 & 90, PGA (0,1)<br>**QoL:** DLQI (0,1)<br>**Safety:** all AEs, nasopharyngitis , upper respiratory tract infection, infection, headache, d/c due to AE)	No<br>**Efficacy:** PASI50, 75 & 90<br>**No QoL**<br>**Safety:** any AE, SAE, ≥grade 3 AE, specific AE, serious infection, d/c due to AE, treatment-related death
**Has there been a review of the literature concerning potential modifiers of treatment effect?**	No discussion	No discussion	No discussion	No discussion	No discussion	Yes, baseline data extracted for age, sex, body weight, psoriasis duration, psoriasis severity, and previous psoriasis treatment that may have acted as effect modifiers	No, expected that potential effect modifiers were balanced across trials due to broadly similar patient characteristics	No discussion	No, adjustments not made to address between-study differences in potential effect modifiers	No discussion	No discussion
**Is there a discussion of the biases to which these trials, or this ensemble of trials, are vulnerable?**	No discussion	Yes, "We did not assess publication bias in this study because it is challenging to do so in a Bayesian NMA and is an area that requires	No discussion	No discussion	No discussion	Yes, application of the Cochrane risk-of-bias tool for randomised trials	Yes, evidence of small study effects favouring older treatments with respect to efficacy outcomes of clear/nearly clear and	Yes, risk of bias rated as low or unclear regarding randomization, blinding, attrition, and reporting bias. "Publication bias against	Yes, heterogeneity of treatment and outcome characteristics assessed as well as study and patient characteristics	No discussion	No discussion

		further research"					PASI75. Suggests evidence of publication bias in favour of small studies showing beneficial effect of established comparators, underestimating newer treatments	null results for PASI75 & 90 outcomes for secukinumab trials compared with other agents"			
**If a bias risk was identified, was any adjustment made to the analysis and was this adequately justified?**	N/A	No	N/A	N/A	N/A	Yes, sensitivity analyses with regard to overall risk of bias reported as comparison-adjusted funnel plot and, in presence of bias, further investigation with meta-regression	No	No	No	N/A	N/A

NICE TSD7 question	Geng et al. 2018[20]	Cameron et al. 2018[21]	Xu et al. 2019[22]	Sawyer et al. 2019a[23]	Sawyer et al. 2019b[24]	Cameron et al. 2019[25]	Bai et al. 2019[26]	Warren et al. 2020a[27]	Warren et al. 2020b[28]	Sbidian et al. 2020[29]	Armstrong et al. 2020[30]
**Has the target population for the decision been clearly defined?**	Yes, adults with moderate-to-severe psoriasis (defined as BSA>10 or PASI >10 and DLQI >10)	Yes, adult patients with moderate-to-severe psoriasis	Yes, patients with moderate-to-severe psoriasis receiving either two of the thirteen studied antibodies and fusion proteins or one of the thirteen antibodies and placebo, average age 40–50 years	Yes, patients (>18 years old) with moderate-to-severe psoriasis	Yes, adults with moderate-to-severe plaque psoriasis	Yes, adults (≥18 years) with moderate-to-severe plaque psoriasis	Yes, patients (>18 years old) with moderate-to-severe psoriasis	Yes, patients (>18 years old) with moderate-to-severe psoriasis	Yes, patients with moderate-to-severe psoriasis	Yes, patients (>18 years old) with moderate-to-severe psoriasis or psoriatic arthritis with concomitant moderate-to-severe psoriasis	Yes, patients with moderate-to-severe psoriasis
**Have all the appropriate treatments in the decision been identified?**	No<br>Adalimumab<br>Briakinumab*<br>Ciclosporin<br>Etanercept<br>Infliximab<br>Methotrexate<br>Ustekinumab	No<br>Adalimumab<br>Apremilast<br>Brodalumab<br>Etanercept<br>Guselkumab<br>Infliximab<br>Ixekizumab<br>Secukinumab<br>Ustekinumab	Yes<br>Adalimumab<br>Alefacept*<br>Briakinumab*<br>Brodalumab<br>Efalizumab*<br>Etanercept<br>Guselkumab<br>Infliximab<br>Itolizumab*<br>Ixekizumab<br>Secukinumab<br>Tildrakizumab<br>Ustekinumab	Yes<br>Adalimumab<br>Apremilast<br>Brodalumab<br>Certolizumab<br>Etanercept<br>Fumaderm/ DMF<br>Guselkumab<br>Infliximab<br>Ixekizumab<br>Risankizumab<br>Secukinumab<br>Tildrakizumab<br>Ustekinumab	Yes<br>Adalimumab<br>Apremilast<br>Brodalumab<br>Etanercept<br>Infliximab<br>Ixekizumab<br>Secukinumab<br>Ustekinumab	Yes<br>Adalimumab<br>Amjevita (ADA biosimilar)<br>Apremilast<br>Brodalumab<br>Etanercept<br>Erelzi (ETN biosimilar)<br>Guselkumab<br>Infliximab<br>Ixekizumab<br>Secukinumab<br>Tildrakizumab<br>Ustekinumab	Yes<br>Brodalumab<br>Guselkumab<br>Ixekizumab<br>Risankizumab<br>Secukinumab<br>Tildrakizumab<br>Ustekinumab	Yes<br>Adalimumab<br>Brodalumab<br>Certolizumab<br>Etanercept<br>Guselkumab<br>Infliximab<br>Ixekizumab<br>Risankizumab<br>Secukinumab<br>Tildrakizumab<br>Ustekinumab	Yes<br>Adalimumab<br>Brodalumab<br>Etanercept<br>Guselkumab<br>Infliximab<br>Ixekizumab<br>Secukinumab<br>Tildrakizumab<br>Ustekinumab	Yes<br>Acitretin<br>Adalimumab<br>Apremilast<br>Bimekizumab*<br>Brodalumab<br>Certolizumab<br>Ciclosporin<br>Deucravacitinib *<br>Etanercept<br>FAEs<br>Guselkumab<br>Infliximab<br>Ixekizumab<br>Methotrexate<br>Mirikizumab*‡<br>Risankizumab<br>Secukinumab<br>Tildrakizumab<br>Tofacitinib*<br>Ustekinumab	Yes<br>Adalimumab<br>Apremilast<br>Brodalumab<br>Certolizumab<br>DMF<br>Etanercept<br>Guselkumab<br>Infliximab<br>Ixekizumab<br>Risankizumab<br>Secukinumab<br>Tildrakizumab<br>Ustekinumab
**Has information been**	Yes	Yes	Yes	Yes<br>EMA-approved doses only	Yes<br>EMA-approved doses only	Yes	Yes	Yes<br>Approved doses only	Yes<br>FDA-approved doses only	Yes	Yes

**provided, how different doses were integrated into the analysis?†**	EMA- and FDA-approved doses only<br>Analysed dosages separately	EMA- and FDA-approved doses only<br>Analysed dosages separately	EMA- and FDA-approved doses<br>Multiple dosages were pooled	Analysed dosages separately	Multiple dosages were pooled	EMA- and FDA-approved doses only<br>Analysed dosages separately	EMA- and FDA-approved doses only<br>Analysed dosages separately	Multiple dosages were pooled	Multiple dosages were pooled	EMA- and FDA-approved doses<br>Multiple dosages were pooled	EMA- and FDA-approved doses only<br>Multiple dosages were pooled
**Have all trials reporting relevant outcomes been included?**	No<br>**Efficacy:** PASI50, 75 & 90<br>**No QoL**<br>**No safety**	No<br>**Efficacy:** PASI90<br>**No QoL**<br>**No safety**	No<br>**Efficacy:** PASI50, 75, 90 & 100, PGA<br>**QoL:** DLQI<br>**Safety:** headache, infection, d/c	No<br>**Efficacy:** PASI50, 75, 90 & 100, NNT for PASI100<br>**No QoL**<br>**No safety**	No<br>**Efficacy:** PASI 75, 90 & 100<br>**No QoL**<br>**No safety**	No<br>**Efficacy:** PASI50, 75, 90 & 100; PGA/IGA (0,1)<br>**QoL:** DLQI (0,1)<br>**Safety:** AE, SAE, d/c due to AE	No<br>**Efficacy:** PASI75 & 100, sPGA/IGA/PGA (0,1)<br>**No QoL**<br>**Safety:** AE, SAE, d/c due to AE	No<br>**Efficacy:** PASI75, 90 & 100<br>**QoL:** DLQI (0,1)<br>**No safety**	No<br>**Efficacy:** PASI75, 90 & 100<br>**No QoL**<br>**No safety**	No<br>**Efficacy:** PASI75 & 90, PGA (0,1)<br>**QoL:** DLQI (0,1)<br>**Safety:** AE, SAE	No<br>**Efficacy:** PASI75, 90 & 100, and respective NNTs<br>**No QoL**<br>**No safety**
**Has there been a review of the literature concerning potential modifiers of treatment effect?**	No discussion	No discussion	No discussion	Yes, discussion of effect of placebo response rates; also found variation in factors like age, baseline PASI, prior exposure to systemic therapy	Yes, prior exposure to biologics thought to be a potential effect modifier; sensitivity analysis was run excluding studies not reporting or in which <5% of patients reported prior biologics experience	No discussion	No discussion	No discussion	No discussion	Yes, baseline demographic and clinical characteristics (age, sex, body weight, duration of psoriasis, severity of psoriasis at baseline, previous psoriasis treatment); however, not enough data available to assess the impact on the results	No discussion
**Is there a discussion of the biases to which these trials, or this ensemble of trials, are vulnerable?**	Yes, application of the Cochrane risk-of-bias tool for randomised trials	Yes, lack of adjustment for cross-trial differences led to different clinical interpretations of the results and tended to bias in favour of treatments that reported lower	Yes, application of the Cochrane risk-of-bias tool for randomised trials	Yes, low risk of bias of included studies in general; only variation in handling of missing data observed	Yes, risk of bias somewhat heterogeneous but most studies rated as having low risk of bias	Yes, application of the Cochrane risk-of-bias tool for randomised trials	Yes, application of the Cochrane risk-of-bias tool for randomised trials	Yes, for some trials, DLQI threshold for NMA inclusion was not met, and thus selection and reporting bias are possible	Yes, application of the Cochrane risk-of-bias tool for randomised trials but no information on bias provided	Yes, application of the Cochrane risk-of-bias tool for randomised trials	No discussion

		placebo response rates									
**If a bias risk was identified, was any adjustment made to the analysis and was this adequately justified?**	Yes, studies with high risk of bias have been excluded from the analysis	Yes, the main aim of the publication was to investigate adjustment for baseline risk (placebo response) versus no response	No	Yes, additional analyses run, (e.g., test for small study bias by) exclusion of trials with <50 patients per arm	No, risk of bias considered low	No	Yes, sensitivity analyses excluding studies with high risk of bias; publication bias investigated with funnel plots	No	No	Yes, sensitivity analyses with exclusion of studies with high risk of bias	N/A

*Indicates treatment that has been withdrawn or not approved for psoriasis.

†Item added by the authors, not part of NICE TSD7 checklist.

‡Not included in any analyses reported in publication.

d/c due to AEs includes discontinuations due to AEs and withdrawals due to AEs.

Study heterogeneity denotes variability in study outcomes that goes beyond what would be expected (or could be explained) due to a measurement error alone.

ADA, adalimumab; AE, adverse event; BSA, body surface area; d/c, discontinuation; DLQI, Dermatology Life Quality Index; DMF, dimethyl fumarate; EMA, European Medicines Agency; ETN, etanercept; FAEs, fumaric acid esters; FDA, Food and Drug Administration; IGA, Investigator's Global Assessment; N/A, not applicable; NICE TSD7, National Institute for Health and Care Excellence Technical Support Document 7; NMA, network meta-analysis; NNT, number needed to treat; PASI50, 75, 90 & 100; Psoriasis Area and Severity Index 50%, 75%, 90% & 100% reduction from baseline; PGA, Physician's Global Assessment; QoL, quality of life; SAE, serious adverse event; SmPC, Summary of Product Characteristics; sPGA, static Physician's Global Assessment.

In all four AICs, the target population for the decision was clearly defined and appropriate comparators were identified (Table S4). Galván-Banqueri et al. compared the interleukin (IL)-12/23 inhibitor, ustekinumab, with tumour necrosis factor alpha inhibitors, adalimumab and infliximab, using etanercept as the bridge comparator, in 2013.[3] The following year, Schmitt et al. compared adalimumab, etanercept, infliximab, ustekinumab, alefacept, and conventional systemics ciclosporin, fumaric acid esters and methotrexate.[4] More recently in 2018, Papp et al. compared adalimumab with etanercept using placebo as a bridge comparator,[6] and Warren et al. compared two IL-17 inhibitors, ixekizumab and secukinumab, using ustekinumab, etanercept and placebo as bridge comparators.[5] All four AICs analysed data for the induction period based on European Medicines Agency (EMA)-approved doses. Most AICs analysed multiple dose regimens separately as appropriate.

All AICs reported Psoriasis Area and Severity Index 75% and 90% reduction from baseline (PASI75 and PASI90) efficacy outcomes. The two earlier and two later AICs also reported PASI 50% reduction from baseline (PASI50) and PASI 100% reduction from baseline (PASI100) outcomes, respectively. Patient quality of life outcomes were included in two AICs, both published in 2018 (Dermatology life Quality Index [DLQI] (0) and DLQI (0,1)). Minimal safety outcomes were included across the AICs; only Papp et al. 2018 included adverse event (AE) outcomes.[6]

There was no discussion in any AIC publication with regard to potential treatment effect modifiers (e.g., prior treatment) as a result of heterogeneity within the included patient population. Similarly, there was no discussion of the biases to which included trials may be vulnerable in three of the four publications; Schmitt et al. reported use of the Cochrane risk-of-bias tool for randomised trials,[31] but any risks of bias identified, or adjustments made, were not discussed.[4]

Table S4. Comparison of adjusted indirect comparisons with respect to definition of the decision problem based on selected National Institute for Health and Care Excellence Technical Support Document 7 checklist questions (N=4)

NICE TSD7 question	Galván-Banqueri et al. 2013[3]	Schmitt et al. 2014[4]	Warren et al. 2018[5]	Papp et al. 2018[6]
**Has the target population for the decision been clearly defined?**	Yes, patients (>18 years old) with moderate-to-severe psoriasis who failed to respond to or were contraindicated/intolerant to other systemic therapies	Yes, patients (>18 years old) with moderate-to-severe psoriasis (patients with <75% plaque psoriasis were excluded)	Yes, patients (>18 years old) with moderate-to-severe psoriasis for ≥6 months before randomisation	Yes, patients from Phase 3 REVEAL, CHAMPION, M10-114 and M10-315 studies with strictest exclusion criteria across all 4 trials applied
**Have all the appropriate treatments in the decision been identified?**	Yes<br>Adalimumab<br>Infliximab<br>Ustekinumab<br>Bridge comparator – etanercept	Yes<br>Adalimumab<br>Alefacept*<br>Ciclosporin<br>Etanercept<br>FAEs<br>Infliximab<br>Methotrexate<br>Ustekinumab	Yes<br>Ixekizumab<br>Secukinumab<br>Bridge comparators – etanercept, placebo and ustekinumab	Yes<br>Adalimumab<br>Etanercept<br>Bridge comparator – placebo
**Has information been provided, how different doses were integrated into the analysis?†**	Yes<br>EMA-approved doses only<br>Analysed dosages separately but pooled etanercept 25mg twice weekly with 50mg weekly in the bridging arm	Yes<br>EMA-approved doses only<br>Analysed dosages separately	Yes<br>EMA-approved doses only<br>Analysed dosages separately	Yes<br>EMA-approved doses only<br>Analysed dosages separately
**Have all trials reporting relevant outcomes been included?**	Yes<br>**Efficacy:** PASI50, 75 & 90<br>**No QoL**<br>**No safety**	Yes<br>**Efficacy:** PASI50, 75 & 90<br>**No QoL**<br>**No safety**	Yes<br>**Efficacy:** PASI75, 90 & 100<br>**QoL:** DLQI (0,1)<br>**No safety**	Yes<br>**Efficacy:** PASI75, 90 & 100, symptom resolution, lesion resolution and CDC<br>**QoL:** DLQI (0)<br>**Safety:** AEs
**Has there been a review of the literature concerning potential modifiers of treatment effect?**	No discussion	No discussion	No discussion	No discussion
**Is there a discussion of the biases to which these trials, or this ensemble of trials, are vulnerable?**	No discussion	Yes, application of the Cochrane Handbook for Systematic Reviews of Interventions checklist	No discussion	No discussion
**If a bias risk was identified, was any adjustment made to the analysis and was this adequately justified?**	N/A	No	N/A	N/A

*Indicates treatment that has been withdrawn or not approved for psoriasis.

†Item added by the authors, not part of NICE TSD7 checklist.

AEs, adverse events; CDC, complete disease control; CI, confidence interval; DLQI, Dermatology Life Quality Index; EMA, European Medicines Agency; FAEs, fumaric acid esters; N/A, not applicable; NICE TSD7, National Institute for Health and Care Excellence Technical Support Document 7; PASI50, 75, 90 & 100; Psoriasis Area and Severity Index 50%, 75%, 90% & 100% reduction from baseline; QoL, quality of life.

3. Issues specific to network synthesis

Table S5. Comparison of network meta-analysis methods specific to network synthesis based on selected National Institute for Health and Care Excellence Technical Support Document 7 checklist questions (N=22)

NICE TSD7 question	Reich et al. 2012[9]	Lin et al. 2012[10]	Gupta et al. 2014[11]	Messori et al. 2015[12]	Signorovitch et al. 2015[13]	Sbidian et al. 2017[14]	Jabbar-Lopez et al. 2017[15]	Gómez-Garcia et al. 2017[16]	Sawyer et al. 2018[17]	Lv et al. 2018[18]	Loos et al. 2018[19]
**Is the network of evidence based on randomized trials connected?**	No network displayed	Yes	Yes	Yes	Yes	Yes	Yes	Yes	Yes	Yes	No network displayed
**Have adequate checks for inconsistency been made?**	Not described	Not described	Yes, node-splitting analyses applied	Not described	Descriptive considerations	Yes, loop-specific approach and side-splitting approach applied	Yes, no detail provided	Yes, significant inconsistency between indirect and direct PASI75 estimates for: 1) etanercept 50 mg BIW vs. ustekinumab 45 mg Q12W 2) ustekinumab 90 mg Q12W comparisons. Reason: maybe heterogeneity in the severity of disease in the populations across different studies	Yes, inconsistency in direct evidence was assessed using random-effects unrelated mean effects model	Yes, node- splitting analyses applied	Not described
**If inconsistency was detected, what adjustments were made to the analysis, and how was this justified?**	N/A	N/A	No significant inconsistency between direct and indirect evidence	N/A	No discussion (finding: PASI75 response for adalimumab differed from recently published NMAs, Reich et al. 2012 and Lin et al. 2012)	Subgroup analysis and meta-regression	None detected	No	No significant inconsistency was identified in the base case or sensitivity analyses networks	Direct and indirect evidence for each comparison under all outcomes, as well as network results, suggested a significant inconsistency between direct and indirect evidence. Overall, no inconsistency was found for each comparison under all outcomes (all p>0.05), which indicated reliable NMA results	N/A

NICE TSD7 question	Geng et al. 2018[20]	Cameron et al. 2018[21]	Xu et al. 2019[22]	Sawyer et al. 2019a[23]	Sawyer et al. 2019b[24]	Cameron et al. 2019[25]	Bai et al. 2019[26]	Warren et al. 2020a[27]	Warren et al. 2020b[28]	Sbidian et al. 2020[29]	Armstrong et al. 2020[30]
**Is the network of evidence based on randomized trials connected?**	Yes	Yes	Yes	Yes	Yes	Yes	Yes	Yes	Yes	Yes	Yes
**Have adequate checks for inconsistency been made?**	Yes, node-splitting analyses applied	Not described	Yes, node-splitting methods and net heat plots conducted to examine degree of inconsistency	Yes, no detail provided	Yes, inconsistency between direct and indirect effect estimates was assessed for any loops in the evidence network using the two-stage Bucher method	Not described	Yes, inconsistency investigated based on ROR to calculate the absolute difference between direct and indirect evidence	Not described	Not described	Yes, loop-specific approach and side-splitting approach applied	Not described
**If inconsistency was detected, what adjustments were made to the analysis, and how was this justified?**	Yes, effect sizes were pooled using an inconsistency model	N/A	No statistical inconsistency observed	No significant inconsistency was identified in the base case or sensitivity analysis networks	No significant inconsistency was identified	N/A	No significant inconsistency was identified apart from: for PASI75, there was statistical loop inconsistency in the loop containing placebo, ustekinumab 45 mg, and ustekinumab 90mg in the combined results of direct and indirect evidence (ROR = 2 114, 95% CI 1.36–3.28)	N/A	N/A	No important heterogeneity identified; global test for inconsistency was not significant	N/A

A key assumption of NMA is the consistency of direct and indirect evidence. Inconsistency occurs in NMA when the direct and indirect evidence conflict.

BIW, twice a week; CI, confidence interval; PASI75; Psoriasis Area and Severity Index 75% reduction from baseline; N/A, not applicable; NICE TSD7, National Institute for Health and Care Excellence Technical Support Document 7; NMA, network meta-analysis; Q12W, every 12 weeks; ROR, relative odds ratio.

Quality and risk of bias assessment

Mediation was required to resolve the assessment of 11/416 of all items, 4/182 of critical items and 7/234 non-critical items. For all items, critical items and non-critical items, Cohen's Kappa was 0.95 (standard error [SE] 0.015, 95% CI 0.92–0.98, $p<0.001$), 0.95 (SE 0.022, 95% CI 0.91–0.99, $p<0.001$) and 0.94 (SE 0.021, 95% CI 0.90–0.98, $p<0.001$), respectively.

Figure S1. Overall confidence in results of adjusted indirect comparisons and network meta-analyses based on AMSTAR 2 assessment of underlying systematic literature reviews (N=26)

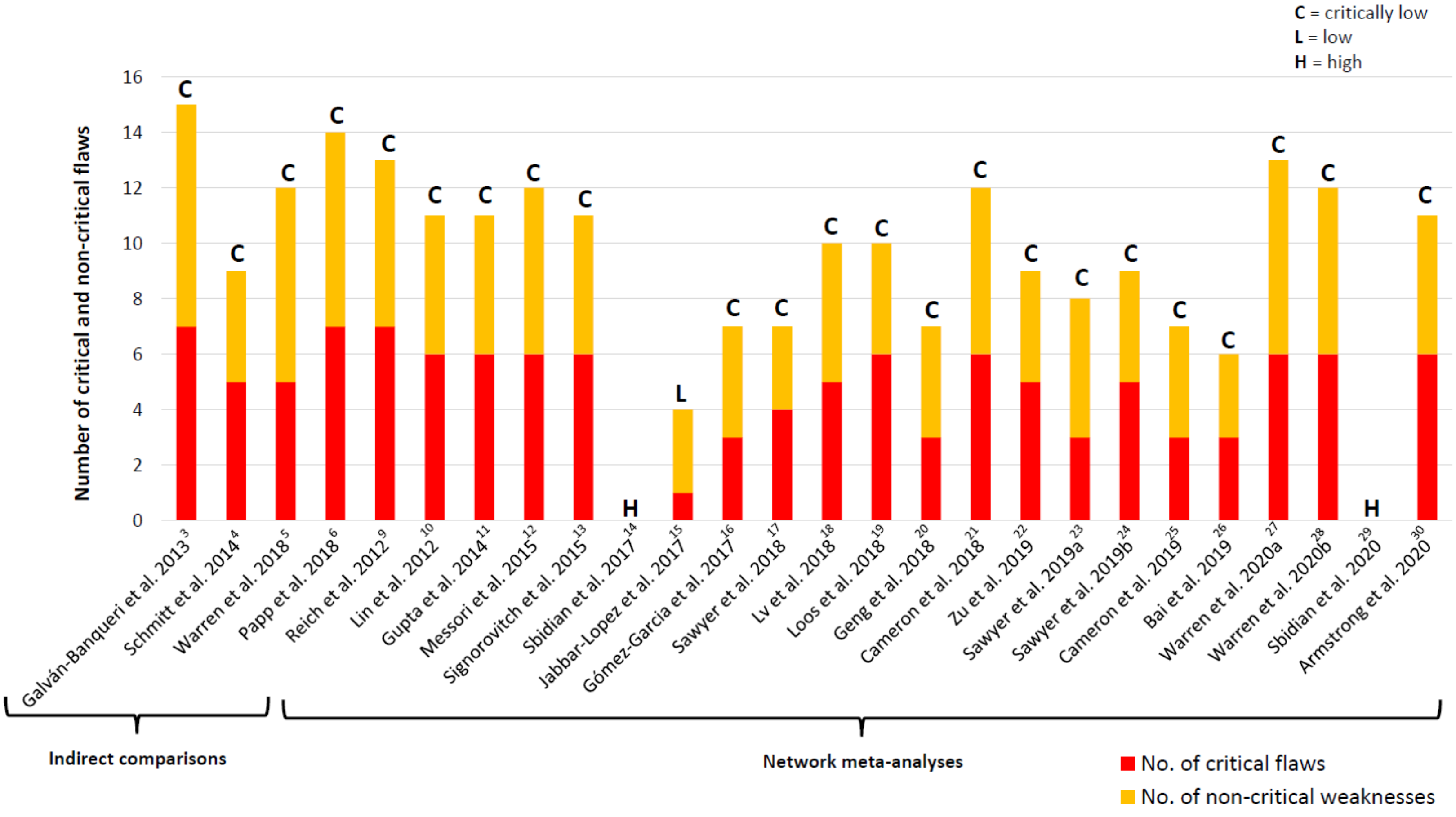

*0 critical flaws, ≤1 non-critical weakness = high confidence in results; 0 critical flaws, >1 non-critical weakness = moderate confidence in results, 1 critical flaw = low confidence in results; >1 critical flaw = critically low confidence in results.

Figure S2. AMSTAR 2 critical and non-critical domain assessment results for systematic literature reviews underlying adjusted indirect comparisons and network meta-analyses (N=26)

a. Seven critical domains

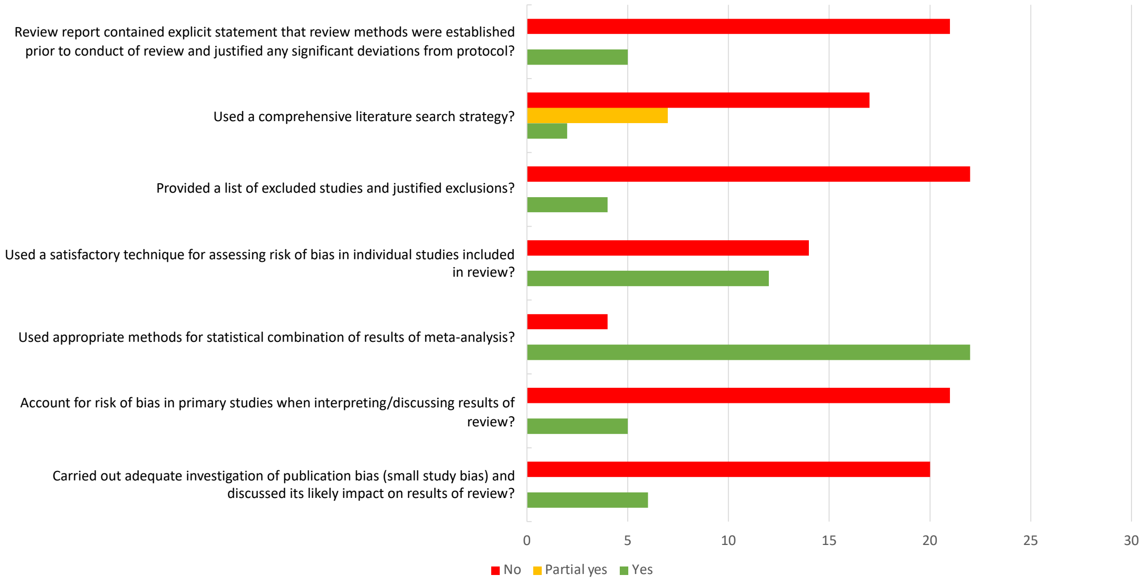

b. Nine non-critical domains

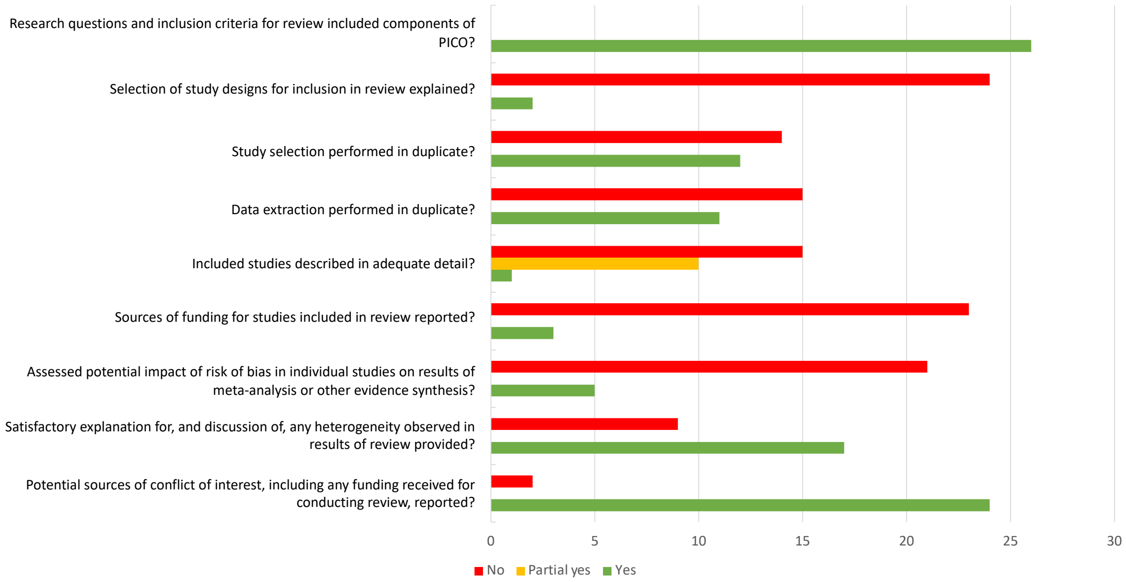

PICO, Patient, population or problem, Intervention, prognostic factor or exposure, Comparison or intervention, Outcome measured or achieved.

References

1. National Institute for Health and Care Excellence (NICE) Decision Support Unit (DSU) Technical Support Document 7: Evidence Synthesis of Treatment Efficacy in Decision Making: a Reviewer's Checklist. 2012. Available from: http://nicedsu.org.uk/wp-content/uploads/2016/03/TSD7-reviewer-checklist.final_.08.05.12.pdf
2. Ranganathan P, Pramesh CS, Aggarwal R. Common pitfalls in statistical analysis: measures of agreement. Perspect Clin Res. 2017;8(4):187–91.
3. Galván-Banqueri M, Marín Gil R, Santos Ramos B, Bautista Paloma FJ. Biological treatments for moderate-to-severe psoriasis: indirect comparison. J ClinPharm Ther. 2013;38(2):121–30.
4. Schmitt J, Rosumeck S, Thomaschewski G, Sporbeck B, Haufe E, Nast A. Efficacy and safety of systemic treatments for moderate-to-severe psoriasis: meta-analysis of randomized controlled trials. Br J Dermatol. 2014;170(2):274–303.
5. Warren RB, Brnabic A, Saure D, Langley RG, See K, Wu JJ, et al. Matching-adjusted indirect comparison of efficacy in patients with moderate-to-severe plaque psoriasis treated with ixekizumab vs. secukinumab. Br J Dermatol. 2018;178(5):1064–71.
6. Papp KA, Yang M, Sundaram M, Jarvis J, Betts KA, Bao Y, et al. Comparison of adalimumab and etanercept for the treatment of moderate to severe psoriasis: an indirect comparison using individual patient data from randomized trials. Value Health. 2018;21(1):1–8.
7. Bucher HC, Guyatt GH, Griffith LE, Walter SD. The results of direct and indirect treatment comparisons in meta-analysis of randomized controlled trials. J Clin Epidemiol. 1997;50:683–91.
8. Signorovitch JE, Wu EQ, Yu AP, Gerrits CM, Kantor E, Bao Y, et al. Comparative effectiveness without head-to-head trials: a method for matching-adjusted indirect comparisons applied to psoriasis treatment with adalimumab or etanercept. Pharmacoeconomics. 2010;28:935–45.
9. Reich K, Burden AD, Eaton JN, Hawkins NS. Efficacy of biologics in the treatment of moderate to severe psoriasis: a network meta-analysis of randomized controlled trials. Br J Dermatol. 2012;166(1):179–88.
10. Lin VW, Ringold S, Devine EB. Comparison of ustekinumab with other biological agents for the treatment of moderate to severe plaque psoriasis: a Bayesian network meta-analysis. Arch Dermatol. 2012;148(12):1403–10.

11. Gupta AK, Daigle D, Lyons DCA. Network meta-analysis of treatments for chronic plaque psoriasis in Canada. J Cutan Med Surg. 2014;18(6):371–8.
12. Messori A, Trippoli S, Fadda V, Maratea D, Marinai C. Subcutaneous biological treatments for moderate to severe psoriasis: interpreting safety data by network meta-analysis. Drugs Real World Outcomes. 2015;2(1):23–7.
13. Signorovitch JE, Betts KA, Yan YS, LeReun C, Sundaram M, Wu EQ, et al. Comparative efficacy of biological treatments for moderate-to-severe psoriasis: a network meta-analysis adjusting for cross-trial differences in reference arm response. Br J Dermatol. 2015;172(2):504–12.
14. Sbidian E, Chaimani A, Garcia-Doval I, Do G, Hua C, Mazaud C, et al. Systemic pharmacological treatments for chronic plaque psoriasis: a network meta-analysis. Cochrane Database Syst Rev. 2017;12(12):CD011535.
15. Jabbar-Lopez ZK, Yiu ZZN, Ward V, Exton LS, Mohd Mustapa MF, Samarasekera E, et al. Quantitative evaluation of biologic therapy options for psoriasis: a systematic review and network meta-analysis. J Invest Dermatol. 2017;137(8):1646–54.
16. Gómez-García F, Epstein D, Isla-Tejera B, Lorente A, Vélez García-Nieto A, Ruano J. Short-term efficacy and safety of new biological agents targeting the interleukin-23-T helper 17 pathway for moderate-to-severe plaque psoriasis: a systematic review and network meta-analysis. Br J Dermatol. 2017;176(3):594–603.
17. Sawyer L, Fotheringham I, Wright E, Yasmeen N, Gibbons C, Holmen Møller A. The comparative efficacy of brodalumab in patients with moderate-to-severe psoriasis: a systematic literature review and network meta-analysis. J Dermatol Treat. 2018;29(6):557–68.
18. Lv J, Zhou D, Wang Y, Zhao J, Chen Z, Zhang J, et al. Quantitative evaluation to efficacy and safety of therapies for psoriasis: a network meta-analysis. Mol Pain. 2018;14:1744806918762205.
19. Loos AM, Liu S, Segel C, Ollendorf DA, Pearson SD, Linder JA. Comparative effectiveness of targeted immunomodulators for the treatment of moderate-to-severe plaque psoriasis: a systematic review and network meta-analysis. J Am Acad Dermatol. 2018;79(1):135–44.e7.
20. Geng W, Zhao J, Fu J, Zhang H, Qiao S. Efficacy of several biological therapies for treating moderate to severe psoriasis: a network meta-analysis. Exp Ther Med. 2018;16(6):5085–95.

21. Cameron C, Hutton B, Druchok C, McElligott S, Nair S, Schubert A, et al. Importance of assessing and adjusting for cross-study heterogeneity in network meta-analysis: a case study of psoriasis. J Comp Eff Res. 2018;7(11):1037–51.

22. Xu G, Xia M, Jiang C, Yu Y, Wang G, Yuan J, et al. Comparative efficacy and safety of thirteen biologic therapies for patients with moderate or severe psoriasis: a network meta-analysis. J Pharmacol Sci. 2019;139(4):289–303.

23. Sawyer LM, Malottki K, Sabry-Grant C, Yasmeen N, Wright E, Sohrt A, et al. Assessing the relative efficacy of interleukin-17 and interleukin-23 targeted treatments for moderate-to-severe plaque psoriasis: a systematic review and network meta-analysis of PASI response. PLoS One. 2019a;14(8):e0220868.

24. Sawyer LM, Cornic L, Levin LÅ, Gibbons C, Møller AH, Jemec GB. Long-term efficacy of novel therapies in moderate-to-severe plaque psoriasis: a systematic review and network meta-analysis of PASI response. J Eur Acad Dermatol Venereol. 2019b;33(2):355–66.

25. Cameron C, Druchok C, Hutton B, McElligott S, Nair S, Schubert A, et al. Guselkumab for the treatment of moderate-to-severe plaque psoriasis during induction phase: a systematic review and network meta-analysis. J Psoriasis Psoriatic Arthritis. 2019;4(2):81–92.

26. Bai F, Li GG, Liu Q, Niu X, Li R, Ma H. Short-term efficacy and safety of IL-17, IL-12/23, and IL-23 inhibitors brodalumab, secukinumab, ixekizumab, ustekinumab, guselkumab, tildrakizumab, and Risankizumab for the treatment of moderate to severe plaque psoriasis: a systematic review and network meta-analysis of randomized controlled trials. J Immunol Res. 2019;2019:2546161.

27. Warren RB, See K, Burge R, Zhang Y, Brnabic A, Gallo G, et al. Rapid response of biologic treatments of moderate-to-severe plaque psoriasis: a comprehensive investigation using Bayesian and frequentist network meta-analyses. Dermatol Ther (Heidelb). 2020a;10(1):73–86.

28. Warren RB, Gooderham M, Burge R, Zhu B, Amato D, Liu KH, et al. Comparison of cumulative clinical benefits of biologics for the treatment of psoriasis over 16 weeks results from a network meta-analysis. J Am Acad Dermatol. 2020b;82(5):1138–49.

29. Sbidian E, Chaimani A, Garcia-Doval I, Do G, Hua C, Mazaud C, et al. Systemic pharmacological treatments for chronic plaque psoriasis: a network meta-analysis. Cochrane Database Syst Rev. 2020;1(1):CD011535.

30. Armstrong AW, Puig L, Joshi A, Skup M, Williams D, Li J, et al. Comparison of biologics and oral treatments for plaque psoriasis: a meta-analysis. JAMA Dermatol. 2020;156(3):Q3 258-69.

31. Higgins JP, Altman DG, Gøtzsche PC, Jüni P, Moher D, Oxman AD, et al. The Cochrane Collaboration's tool for assessing risk of bias in randomised trials. BMJ. 2011;343:d5928.
